# Incidence, Prediction, and Outcomes of Major Bleeding After Percutaneous Coronary Intervention in Chinese Patients

**DOI:** 10.1016/j.jacasi.2021.12.009

**Published:** 2022-04-26

**Authors:** Andrew Kei-Yan Ng, Pauline Yeung Ng, April Ip, Ian Wood-Hay Ling, Lap-Tin Lam, Chung-Wah Siu

**Affiliations:** a Cardiac Medical Unit, Grantham Hospital, Hong Kong SAR, China; b Department of Adult Intensive Care, Queen Mary Hospital, Hong Kong SAR, China; c Division of Respiratory and Critical Care Medicine, Department of Medicine, Li Ka Shing Faculty of Medicine, The University of Hong Kong, Hong Kong SAR, China; d Department of Medicine, Queen Mary Hospital, The University of Hong Kong SAR, China

**Keywords:** all-cause mortality, major adverse cardiac events, major bleeding, myocardial infarction, percutaneous coronary intervention, repeat revascularization, stroke, AUC, area under the curve, DAPT, dual antiplatelet therapy, eGFR, estimated glomerular filtration rate, MACE, major adverse cardiac events, MB, major bleeding, PCI, percutaneous coronary intervention

## Abstract

**Background:**

The patterns of late major bleeding (MB) after percutaneous coronary intervention (PCI) remain unknown in Chinese patients.

**Objectives:**

This study sought to determine the incidence, prediction, and long-term outcomes of late MB in Chinese patients.

**Methods:**

This was a retrospective cohort study from 14 hospitals in Hong Kong. Participants were patients undergoing first-time PCI without MB within 30 days or death within 1 year. Patients were stratified by the presence of late MB, defined as MB between 30 and 365 days. The primary endpoint was all-cause mortality. The secondary endpoints were major adverse cardiac events (MACE).

**Results:**

A total of 32,057 patients were analyzed. After adjustment for baseline characteristics, periprocedural characteristics, and medications on discharge, the risks of all-cause mortality at 5 years were significantly higher with late MB (HR: 2.15; 95% CI: 1.92-2.41; *P <* 0.001). Late MB was also associated with a higher risk of MACE (HR: 1.57; 95% CI: 1.03-1.50; *P <* 0.001), myocardial infarction (HR: 1.25; 95% CI: 1.04-1.52; *P =* 0.02), and stroke (HR: 1.38; 95% CI: 1.09-1.73; *P =* 0.006). The CARDIAC (anti-Coagulation therapy, Age, Renal insufficiency, Drop In hemoglobin, baseline Anemia in Chinese patients) score had a good discriminating power for prediction of MB within 365 days (area under the receiver-operating characteristic curve: 0.76).

**Conclusions:**

Late MB was independently associated with a higher risk of mortality, MACE, myocardial infarction, and stroke in patients undergoing PCI. The CARDIAC score is a simple model that can predict MB after PCI. Prevention of MB represents an important strategy to optimize cardiovascular outcomes for patients undergoing PCI.

Major bleeding (MB) occurs in up to 8% of patients undergoing percutaneous coronary intervention (PCI) and has a strong association with subsequent mortality and adverse coronary outcomes.^1^, ^2^, ^3^, ^4^, ^5^ Prognostically, it can be as deleterious as myocardial infarction.^1^^,^^3^ Not only potentially fatal, bleeding is also associated with excess mortality beyond 30 days.^2^^,^^4^ Benefits of intensification or extension of antithrombotic therapy after PCI were counterbalanced by bleeding-related harm.^6^^,^^7^ Known as the East Asian paradox, East Asians have different thrombotic and bleeding profiles from White patients, with a higher vulnerability to bleeding and lower susceptibility to ischemic events.^8^, ^9^, ^10^, ^11^ Unfortunately, even though East Asians are the largest ethnic group (>1.6 billion in population) and half of those undergoing PCI are considered at high bleeding risk,^12^^,^^13^ the impacts of MB were derived from studies that East Asians were largely underrepresented. Therefore, association between MB and subsequent long-term outcomes in East Asians remains unexplored.

Prevent of bleeding during a late period (approximately 1-12 months after PCI) has been utilized as a therapeutic window to improve clinical outcomes, as antithrombotic therapy can be safely curtailed. Examples include shorter dual antiplatelet therapy (DAPT),^14^, ^15^, ^16^ de-escalation to a less potent P2Y_12_ inhibitor,^17^ and omission of aspirin in those receiving oral anticoagulation therapy.^18^ These strategies are contingent upon associations between late MB and adverse outcomes, as well as reliability of bleeding risk score predication. Because such data on East Asian populations were sparse, we sorted to determine the incidence, prediction, and long-term outcomes of late MB in a territory wide cohort of patients receiving PCI in Hong Kong.

## Methods

### Study population and design

Data from all patients who underwent first-time PCI between January 1, 2004, and December 31, 2017, from all 14 government funded hospitals that performed PCI and recorded in a territory-wide PCI registry were reviewed. Patients’ baseline characteristics, exposures, and outcomes were retrieved from the PCI Registry and Clinical Data and Analysis Reporting System. This system was capable of capturing virtually all diagnosis coding, laboratory findings, and blood transfusions within all public hospitals in Hong Kong. We included all adult patients (18 years of age or older) of Chinese ethnicity who underwent first-time PCI. Exclusion criteria were patients who died before hospital discharge. Patients with MB within 30 days after PCI were included in the estimation of MB risk after PCI but were excluded in the subsequent analysis on the association between late MB and clinical outcomes. The study was approved by the Institutional Review Board of the University of Hong Kong/Hospital Authority.

### Definitions of exposure and outcome variables

MB was defined as a composite of fatal bleeding events, bleeding that occurred in the critical sites (intracranial, intra-articular, or intramuscular with compartment syndrome, intraocular, pericardial, retroperitoneal), bleeding necessitating transfusion of ≥2 U of blood product, or bleeding that caused a drop in hemoglobin of ≥2g/dL, in accordance to the International Society on Thrombosis and Haemostasis.^19^ Late MB was defined as MB that occurred between 30 and 365 days after PCI. This time window was justified by the need to focus only on late events occurring in patients already stabilized post-PCI, excluding early events that are largely influenced by index clinical presentation and PCI, in reference to similar studies.^4^^,^^20^ This also coincides with the time window that shortening or de-escalation of DAPT can be safely considered.

The primary endpoint was all-cause mortality, as a time-to-first-event analysis starting from 365 days and up to 5 years after PCI. The secondary endpoints were major adverse cardiac events (MACE), defined as a composite outcome of all-cause mortality, nonfatal myocardial infarction, stroke and any unplanned coronary revascularization, and individual components of MACE except all-cause mortality, over the same observation period as the primary endpoint.

### Statistical analysis

All analyses were performed with prespecified endpoints and statistical methods. Unadjusted analyses were made using chi-square tests for categorical variables, Student’s *t* test or Wilcoxon rank sum tests for continuous variables, and time-to-first-event analysis for estimation of incidence rate. Cox regression analysis was performed to evaluate the independent relationship between late MB and clinical outcomes, adjusting for potential confounders selected a priori based on published data and biological plausibility. Variables adjusted were sex, age, tobacco use, diabetes mellitus, hypertension, dyslipidemia, cerebrovascular disease, peripheral vascular disease, chronic obstructive pulmonary disease, peripheral artery disease, prior myocardial infarction, prior coronary artery bypass surgery, congestive heart failure, atrial fibrillation or flutter, chronic kidney disease with estimated glomerular filtration rate (eGFR) <60 mL/min/1.73 m^2^, baseline anemia (hemoglobin <13 g/dL for men, <12 g/dL for women), history of cancer, acute coronary syndrome, number of epicardial arteries affected, and medications on discharge (aspirin, potent P2Y_12_ inhibitor [ie, ticagrelor or prasugrel], anticoagulation therapy, beta-blocker, angiotensin blockade, proton pump inhibitor).

### Sensitivity analyses

To account for changes in medical practice and treatment over time, we performed additional sensitivity analysis using a mixed-effects model to adjust for the calendar year in which PCI was performed.

To assess for any residual confounding by treatment selection, we performed falsification testing with new diagnosis of cancer and hip fracture after PCI. Cancer and hip fracture were selected based on their association with mortality but were biologically unlikely to be causally related to late MB.^21^^,^^22^

In the primary analysis, the complete case method was adopted to address missing data. To test the robustness of our results, the regression analysis was repeated with the entire cohort using the technique of multiple imputations by chained equations to account for missing data.

### Exploratory analyses

We explored the time-varying effects of late MB on all-cause mortality with a landmark analysis. The outcome were examined separately between 1 to 3 years, 3 to 5 years, 1 to 5 years, and 1 year to last follow-up after PCI using the same regression model in the primary analysis.

We used our Chinese cohort to externally validate the performance of the ADAPT (Asian Dual Anti-Platelet Therapy) bleeding risk score, developed in a Korean cohort, to predict MB within 3 years after PCI.^23^ Next, we developed a risk score to predict the occurrence of MB within 1 year after PCI. All patients were randomly divided in 1:1 ratio into development and validation cohorts. Backward stepwise logistic regression analysis was used to identify strongest risk factors of MB, and a probability value threshold of 10% was used in the selection model building process. The area under the curve (AUC) of the receiver-operating characteristic was used to evaluate the model discrimination between patients with and without MB.

Data management and statistical analyses were performed in Stata software, version 16 (StataCorp). A 2-tailed *P* value of <0.05 was considered statistically significant.

## Results

### Patients and characteristics

**Figure 1:**
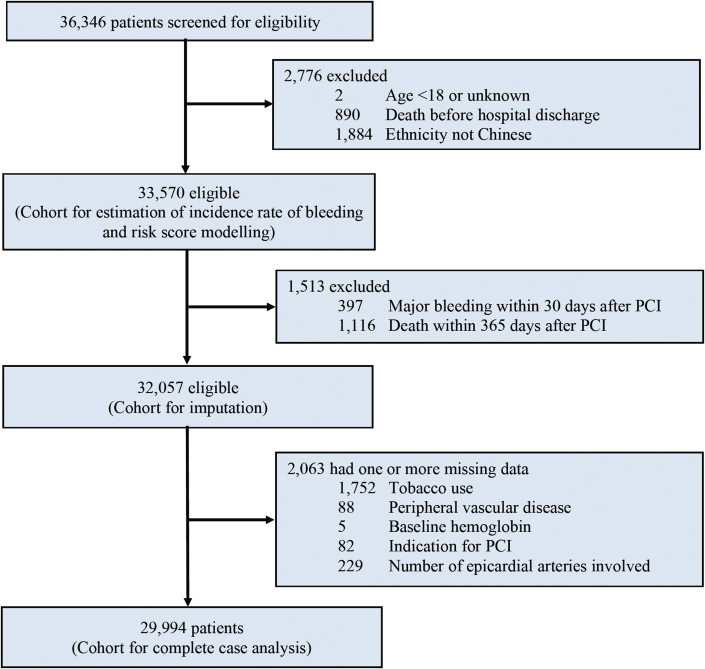
Study Profile PCI = percutaneous coronary intervention.

Between January 2004 and December 2017, a total of 36,346 patients were considered for inclusion: 2,776 were excluded caused by any of the following exclusion criteria—age younger than 18 years or unknown, death before hospital discharge after PCI, or ethnicity not Chinese. The remaining 33,570 patients were included in the estimation for incidence rate of MB and risk score modeling. A further 1,513 patients were excluded for outcome analysis of late MB caused by MB occurring within 30 days or death within 365 days after PCI. A total of 2,063 (6.1%) patients were excluded from the complete case analysis caused by missing values in any of the variables used in the Cox regression model (Figure 1).

### Incidence rate of MB

**Figure 2:**
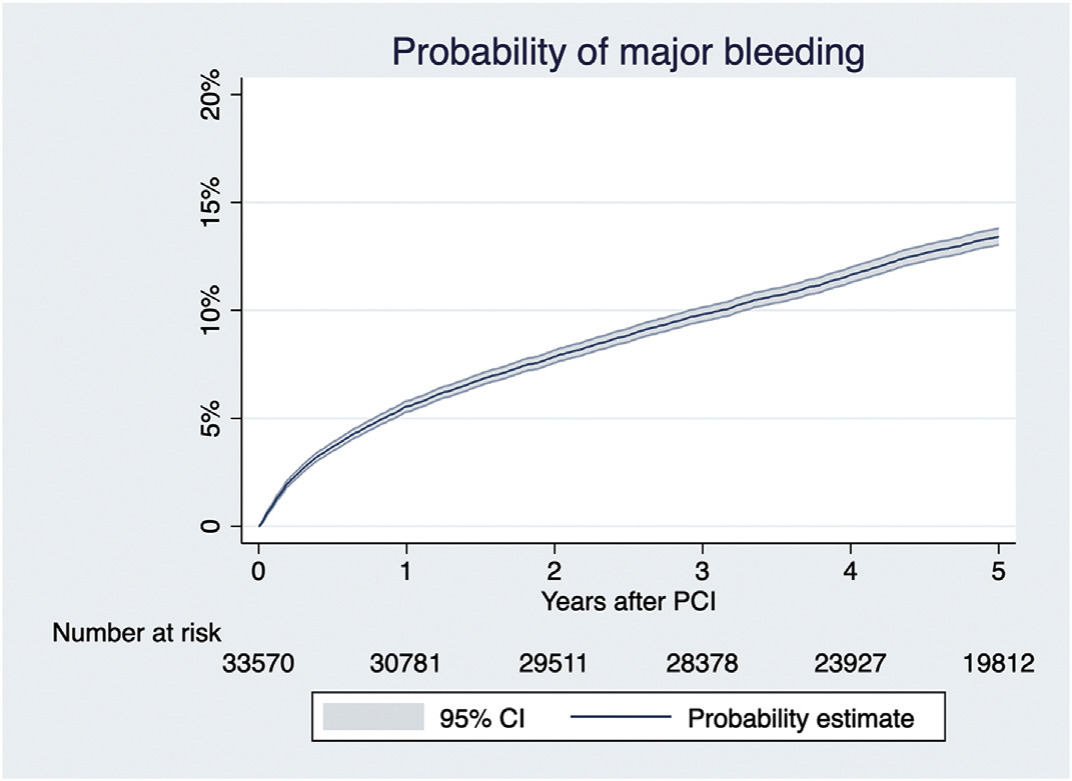
Estimated Probability of Major Bleeding The estimated probability of major bleeding up to 5 years. PCI = percutaneous coronary intervention.

The estimated probability of MB is shown in Figure 2. MB occurred in 1,808 (5.4%) patients between hospital discharge and 1 year after PCI, corresponding to an annualized rate of 5.74% (95% CI: 5.48%-6.01%). The types of bleeding are presented in Supplemental Table 1. Of the remaining patients, MB occurred in 2,348 (7.6%) patients between 1 and 5 years after PCI, corresponding to an annualized rate of 2.19% (95% CI: 2.11%-2.48%).

Among the 29,994 patients who entered the primary analysis model, 561 (1.9%) patients had late MB and 29,722 (98.1%) patients had no late MB. Patients in the late MB group were more frequently female, were older, and had more comorbidities including diabetes, hypertension, cerebrovascular disease, peripheral artery disease, congestive heart failure, and atrial tachyarrhythmia. Patients in the late MB group had more laboratory test abnormalities, including reduced eGFR and hemoglobin, and were also more frequently prescribed anticoagulation and proton pump inhibitor therapy. Table 1 shows the baseline and characteristics of the study population.

**Table 1:** Baseline Characteristics of Patients

	Late MB (n = 1,193)	No Late MB (n = 28,801)	*P* Value
Female	376 (31.5)	6,914 (24.0)	<0.001
Age, y	68.6 ± 10.9	64.4 ± 11.3	<0.001
Age >75 y	377 (31.6)	5,742 (19.9)	<0.001
Tobacco use	470 (39.4)	13,472 (46.8)	<0.001
Diabetes mellitus	565 (47.4)	10,061 (34.9)	<0.001
Hypertension	922 (77.3)	18,239 (63.3)	<0.001
Dyslipidemia	737 (61.8)	18,386 (63.8)	0.15
Cerebrovascular disease	180 (15.1)	2,565 (8.9)	<0.001
Chronic obstructive pulmonary disease	35 (2.9)	701 (2.4)	0.27
Peripheral artery disease	53 (4.4)	352 (1.2)	<0.001
Prior myocardial infarction	213 (17.9)	3,622 (12.6)	<0.001
Prior coronary artery bypass grafting	21 (1.8)	448 (1.6)	0.58
Congestive heart failure	193 (16.2)	2,104 (7.3)	<0.001
Atrial fibrillation or flutter	111 (9.3)	1,354 (4.7)	<0.001
Chronic kidney disease (eGFR <60 mL/min/1.73 m^2^)	484 (40.6)	4,983 (17.3)	<0.001
Anemia at baseline^a^	679 (56.9)	8,221 (28.5)	<0.001
History of cancer	103 (8.6)	1,331 (4.6)	<0.001
Acute coronary syndrome	974 (81.6)	22,982 (79.8)	0.12
Number of epicardial arteries affected			<0.001
1 vessel	446 (37.4)	13,046 (45.3)	
2 vessels	406 (34.0)	9,665 (33.6)	
3 vessels	341 (28.6)	6,090 (21.1)	
Aspirin on discharge	1,172 (98.2)	27,988 (97.2)	0.029
P2Y_12_ inhibitor on discharge	1,175 (98.5)	28,443 (98.8)	0.42
Potent P2Y_12_ inhibitor on discharge	100 (8.4)	3,141 (10.9)	0.006
Anticoagulation on discharge	81 (6.8)	858 (3.0)	<0.001
Beta-blocker on discharge	932 (78.1)	21,245 (73.8)	<0.001
Angiotensin blockade on discharge	842 (70.6)	19,245 (66.8)	0.007
Statin on discharge	1,053 (88.3)	26,027 (90.4)	0.016
Proton pump inhibitor on discharge	668 (56.0)	14,033 (48.7)	<0.001

Values are n (%) or mean ± SD.

eGFR = estimated glomerular filtration rate. MB = major bleeding.

a Hemoglobin <13 g/dL for men, <12 g/dL for women.

### Primary outcome

During the observation period, the primary outcome of all-cause mortality developed in 349 (29.3%) and 2,553 (8.9%) patients in the late MB group and no late MB group, respectively, corresponding to an absolute risk difference of 20.4% (95% CI: 17.8%-23.0%). The estimated annualized mortality rates were 9.50% (95% CI: 8.56%-10.56%) for those with late MB and 2.47% (95% CI: 2.38%-2.57%) for those without MB (Central Illustration). In the adjusted analysis, the risks of mortality were significantly higher with late MB (HR: 2.15; 95% CI: 1.92-2.41; *P <* 0.001) (Table 2).

**Central Illustration:**
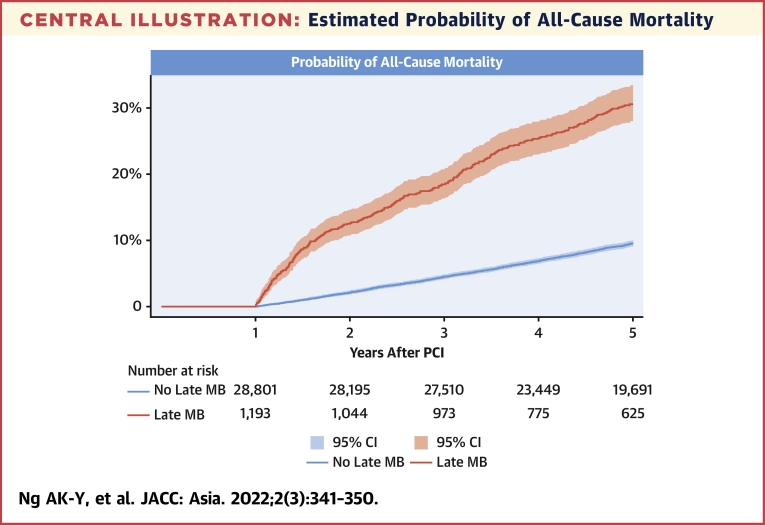
Estimated Probability of All-Cause Mortality The unadjusted probability of all-cause mortality was higher in the late major bleeding (MB) group (log-rank *P <* 0.001). PCI = percutaneous coronary intervention.

**Table 2:** Annualized Incidence Rate and Adjusted Hrs of Primary and Secondary Outcomes

	Annualized Incidence Rate (95% CI) (%)		HR (95% CI)	*P***Value**
	Late MB Group	No Late MB Group		
Primary				
All-cause mortality	9.50 (8.56-10.56)	2.47 (2.38-2.57)	2.15 (1.92-2.41)	<0.001
Cardiovascular mortality	2.84 (2.35-3.42)	0.76 (0.71-0.81)	1.88(1.52-2.33)	<0.001
Secondary				
Major adverse cardiac events	13.18 (11.76-14.77)	5.79 (5.63-5.95)	1.57 (1.40-1.77)	<0.001
Myocardial infarction	3.96 (3.30-4.76)	2.21 (2.12-2.31)	1.25 (1.04-1.51)	0.020
Unplanned revascularization	2.12 (1.65-2.71)	1.81 (1.73-1.90)	1.05(0.82-1.36)	0.684
Stroke	2.50 (2.01-3.11)	1.23 (1.16-1.29)	1.38 (1.09-1.73)	0.006
Ischemic stroke	1.78 (1.39-2.26)	0.95 (0.90-1.01)	1.27(0.98-1.65)	0.075
Hemorrhagic stroke	0.88 (0.62-1.25)	0.35 (0.32-0.39)	1.72(1.18-2.50)	0.004

MB = major bleeding.

**Table 3:** Adjusted HRs of All-Cause Mortality at Various Landmarks

	Annualized Rate (95% CI) (%)		HR (95% CI)	*P* Value
	Late MB Group	No Late MB Group		
Time after PCI				
1-3 y	10.44 (9.15-11.92)	2.29 (2.17-2.42)	2.41 (2.08-2.80)	<0.001
3-5 y	8.24 (6.93-9.79)	2.69 (2.54-2.84)	1.81 (1.51-2.18)	<0.001
5 y until last follow-up	7.59 (6.56-8.79)	3.71 (3.58-3.84)	1.32 (1.14-1.54)	<0.001
1 y until last follow-up	8.75 (8.04-9.53)	3.04 (2.96-3.11)	1.80 (1.64-1.97)	<0.001

PCI = percutaneous coronary intervention.

### Secondary outcomes

Late MB was associated with higher risk of MACE (HR: 1.57; 95% CI: 1.40-1.77; *P <* 0.001), myocardial infarction (HR: 1.25; 95% CI: 1.04-1.51; *P =* 0.020), and stroke (HR: 1.38; 95% CI: 1.09-1.73; *P =* 0.006). The risk of unplanned revascularization was not significantly different across groups (Table 2).

### Sensitivity Analyses

After adjustment of calendar year that PCI was performed, the late MB-mortality association remained significant (HR: 2.10; 95% CI: 1.88-2.37; *P* < 0.001), consistent with the primary analysis. Late MB was also associated with higher risks of all secondary outcomes (Supplemental Table 2).

Falsification testing showed that late MB was not associated with new cancer diagnosis (HR: 0.91; 95% CI: 0.66-1.23; *P =* 0.562) and hip fracture (HR: 1.70; 95% CI: 0.92-3.11; *P =* 0.090), suggesting no significant residual confounding.

A total of 5 variables in the Cox regression model had missing data (Figure 1). To address the issue with missing data, multiple imputation by chained equation was conducted. The imputed cohort included all 2,063 (6.1%) patients who were excluded caused by missing values in any of the variables used in the model. In the imputed data set, the risks of all-cause mortality were significantly higher with late MB (HR: 2.17; 95% CI: 1.94-2.42; *P <* 0.001), consistent with the complete case cohort.

### Exploratory analyses

In landmark analysis, the excess mortality risk associated with late MB was strongest between 1 and 3 years, became less strong between 3 and 5 years, but remained significant even after 5 years (Table 3).

**Table 4:** Calculation of CARDIAC Score for Prediction of Major Bleeding

	Score	Range
Anticoagulation therapy on discharge	2	0-2
Age		0-4
<50 y	0	
50-59 y	1	
60-69 y	2	
70-79 y	3	
80 y or above	4	
Renal insufficiency		0-4
eGFR >60 mL/min/1.73 m^2^	0	
eGFR 45-60 mL/min/1.73 m^2^	1	
eGFR 30-45 mL/min/1.73 m^2^	2	
eGFR 15-30 mL/min/1.73 m^2^	3	
eGFR <15 mL/min/1.73 m^2^	4	
Drop in hemoglobin		
Every g/dL below baseline **value**, rounded to the nearest integer using the lowest hemoglobin during hospital stay for PCI	1 per g/dL drop	Lowest 0, highest 10 in our cohort
Anemia at baseline		
Every g/dL below 12 g/dL of baseline hemoglobin, rounded to the nearest integer	1 per g/dL below 12 g	Lowest 0, highest 8 in our cohort
Total score		0-16 in our cohort

The optimum cutoff was ≥5.

CARDIAC = anti-Coagulation therapy, Age, Renal insufficiency, Drop In hemoglobin, baseline Anemia in Chinese patients; eGFR = estimated glomerular filtration rate; PCI = percutaneous coronary intervention.

External validation of the ADAPT bleeding risk score with our cohort showed moderate discriminating power for prediction of MB at 3 years in our cohort (AUC: 0.71; 95% CI: 0.70-0.72). The sensitivity and specificity were 59% and 72%, respectively, in our cohort at the recommended cutoff of >3 points.^23^

**Figure 3:**
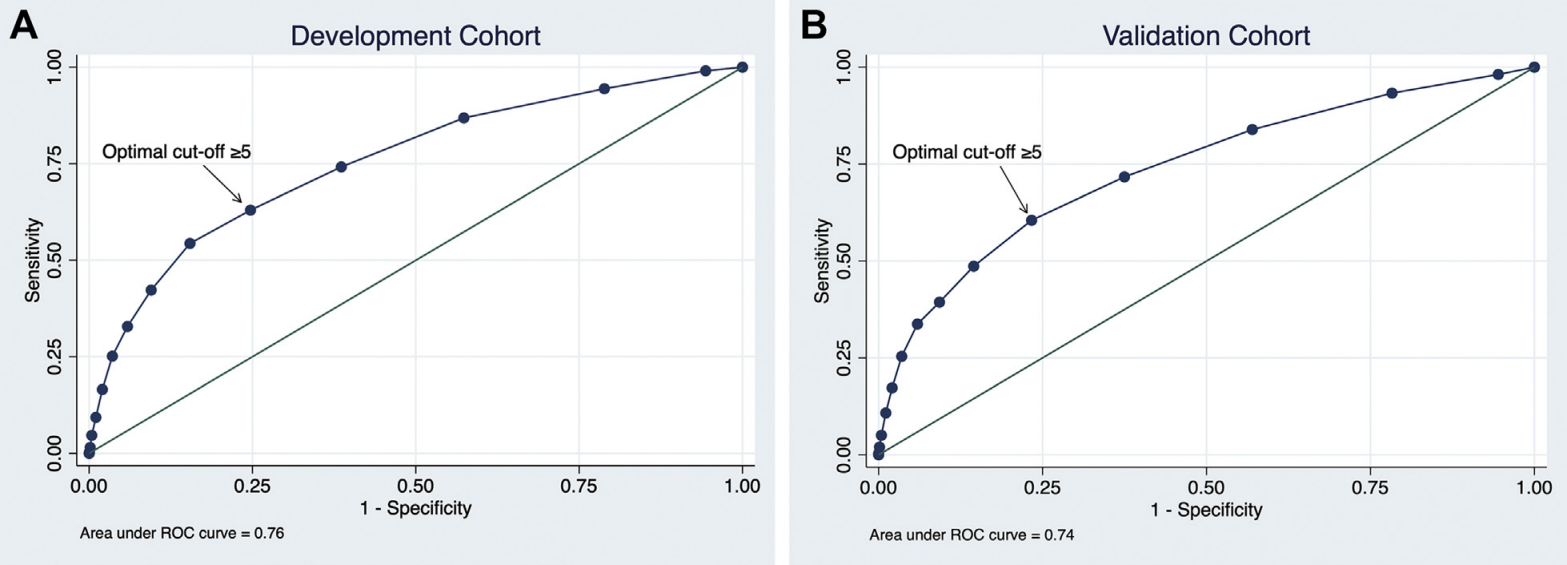
ROC of the Risk Score Receiver-operating characteristic (ROC) of the CARDIAC (anti-Coagulation therapy, Age, Renal insufficiency, Drop In hemoglobin, baseline Anemia in Chinese patients) risk score to predict major bleeding between hospital discharge and 1 year in the development and validation cohorts. The optimal cutoff was ≥5 points.

We developed a new risk score for prediction of MB between hospital discharge and 1 year. Five variables were included into the final logistic regression model: anticoagulation therapy on discharge, age, eGFR, drop in hemoglobin after PCI, and baseline anemia (Supplemental Table 3). In the development cohort with an overall MB risk of 5.4%, the discriminating power of the new risk score was good (AUC: 0.76; 95% CI: 0.74-0.77; *P <* 0.001). The optimal cutoff for prediction of MB was ≥5 points, conferring a sensitivity of 63% and specificity of 75% (Figure 3). In the validation cohort with an overall MB risk of 5.4%, the risk score retained its discriminating power (AUC: 0.74; 95% CI: 0.72-0.76; *P <* 0.001; sensitivity of 61% and specificity of 77% at optimal cutoff) (Figure 3). The risk score is referred to as the CARDIAC (anti-Coagulation therapy, Age, Renal insufficiency, Drop In hemoglobin, baseline Anemia in Chinese patients) score. The score calculation is detailed in Table 4. The calibration plot and absolute risk of MB are detailed in Supplemental Figure 1 and Supplemental Table 4.

## Discussion

In this territory-wide PCI registry with Chinese patients exclusively, the estimated incidence rate of MB after PCI was approximately 5% in the first year and 2% thereafter. Late MB was significant associated with long-term mortality up to 5 years after PCI, and the association remained consistent after adjustment for potential confounding factors and multiple sensitivity analyses. A simple prediction model was able to predict MB within the first year after PCI.

The incidence rate of MB in our cohort was higher than other Western cohorts (approximately 1%-3% within first year)^13^ but was similar to other East Asian registries.^12^^,^^24^^,^^25^ This was probably related to the East Asian paradox and a high proportion of patients with HBR in East Asian countries.^8^^,^^10^^,^^12^

The bleeding-mortality relationship in our study is similar to previous findings in Western populations. Early post-procedural bleeding events are associated with 3-fold increase in long-term mortality.^26^ The excess risk persists up to 6 years and monotonically accrues over time.^27^^,^^28^ Post-discharge (ie, non–procedure related) bleeding events have also been associated with a 2- to 7-fold increase in long-term mortality.^1^, ^2^, ^3^, ^4^, ^5^ Potential mechanisms of bleeding-related deaths are direct bleeding related fatality, thrombotic complications resulting from prothrombotic effects of bleeding or transfusion of blood products, interruption of DAPT, and overlapping risk factors.^2^^,^^29^ Because our cohort comprised bleeding survivors only and we evaluated for long-term outcomes, the excess mortality observed cannot be remedied by successful treatment for bleeding. Furthermore, although the excess mortality risk gradually declined 3 years post-PCI as a result of the survivor effect and diminishing impact from remote events,^4^ the excess risk was still detectable after 5 years. These time patterns highlight the importance of prevention. Evidence-based strategies include shorter DAPT duration, de-escalation of potent P2Y_12_ inhibitors, and ulcer prophylaxis with proton pump inhibitors.^14^, ^15^, ^16^, ^17^^,^^30^

The relationship between late MB and long-term outcomes was mostly derived from European and American cohorts.^2^^,^^3^ However, East Asians are known to have different thrombotic and bleeding profiles than White patients.^8^, ^9^, ^10^, ^11^ This may lead to a differential ischemia-bleeding trade-off in East Asians, and major international guidelines have called for more research on the field.^13^^,^^31^^,^^32^ From a large registry in the United States, Asians with ST-segment elevation myocardial infarction had a higher risks of in-patient MB than White patients but without a significant difference in in-hospital mortality.^33^ A cohort study from Taiwan showed that MB after PCI was associated with excess 1-year mortality and MACE.^34^ The excess mortality concentrated in the early period after bleeding, suggestive of direct causation. Our study provided new evidence on the robust association and risk estimates between MB and its long-term sequelae in East Asians. Because the late MB-mortality relationship has not been clearly delineated in East Asian populations until now, guidelines have provided inconsistent recommendations in the management for East Asians at high bleeding risk.^31^^,^^35^ Our findings will be valuable to develop more precise and evidence based guideline recommendations.

Currently available bleeding risk scores have moderate accuracy in prediction of MB (AUC: 0.64-0.73).^13^ Bleeding models developed in Western populations tend to underestimate bleeding risk in East Asian populations.^36^ An Asian-specific scoring system to predict MB after PCI has been called for.^13^^,^^35^ The ADAPT score, developed from Korean patients for the prediction of MB within 3 years, had a fair performance (AUC: 0.63-0.67) in the original publication.^23^ External validation with our cohort yielded similar performance. The CREDO-Kyoto bleeding risk score developed and validated in Japanese patients had a similar performance as well (AUC: 0.66).^24^ The CARDIAC scoring system developed in the current study has a good discriminating power for MB at 1 year. It also has the advantages of reliance on only a few clinically readily available variables, not requiring complex calculation with a Web calculator (eg, PRECISE-DAPT [PREdicting bleeding Complications In patients undergoing Stent implantation and subsEquent Dual Antiplatelet Therapy] risk score).^37^ It can serve to identify Chinese patients at high bleeding risk for tailored therapy in routine clinical practice.

### Study Limitations

This study had some limitations. First, the observational nature of the study conferred risks of unmeasured confounding and bias, but we had adjusted extensively by the Cox regression model for potential confounders, and the findings were consistent in multiple sensitivity analyses. Nonetheless, the impact of MB, by nature, cannot be studied in a randomized setting. Second, this study included only Chinese patients and may not be generalizable across other ethnic groups. Third, our study described the MB-mortality relationship in bleeding survivors only. The overall MB-mortality association is likely to be even stronger if direct bleeding fatality is also considered.

## Conclusions

Late MB was independently associated with a higher risk of mortality, MACE, myocardial infarction, and stroke in patients undergoing PCI. The CARDIAC score is a simple model that can predict of MB after PCI. Prevention of MB represents an important strategy to optimize cardiovascular outcomes for patients undergoing PCI.

Perspectives **COMPETENCY IN PATIENT CARE AND PROCEDURAL SKILLS:** In Chinese patients undergoing PCI, MB is related to long-term mortality, MACE, myocardial infarction, and stroke. The CARDIAC score is a novel scoring system that can aid prediction of MB.

**TRANSLATIONAL OUTLOOK:** Prevention of MB represents an important opportunity to improve clinical outcomes for Chinese or Asian patients undergoing PCI.

## Funding Support and Author Disclosures

The authors have reported that they have no relationships relevant to the contents of this paper to disclose.
