# Supplementary material for: Incidence, Prediction, and Outcomes of Major Bleeding After Percutaneous Coronary Intervention in Chinese Patients
Source: JACC Asia. 2022 Apr 26;2(3):341–50. doi: 10.1016/j.jacasi.2021.12.009 (PMC9627816; doi:10.1016/j.jacasi.2021.12.009)
Supplement: Supplemental Data [file mmc1.docx]

**Supplementary Appendix**

**ENDPOINT DEFINITIONS**

**Death**

Death is classified as cardiovascular or non-cardiovascular. The cause of death will be determined by the principal condition that resulted in the death, not the immediate mode of death. Managing physicians will utilize all available information provided, along with clinical expertise, in their adjudication of the cause of death.

**Stroke**

Stroke is defined as an acute episode of focal or global neurological dysfunction caused by cerebral vascular injury as a result of infarction or hemorrhage not caused by trauma. Ischemic stroke is defined as an acute episode of focal cerebral dysfunction caused by cerebral infarction. Hemorrhagic stroke is defined as an acute episode of focal or global cerebral dysfunction caused by intraparenchymal, intraventricular, or subarachnoid hemorrhage not caused by trauma. Subdural hematomas are intracranial hemorrhage events but not strokes.

**Intracranial hemorrhage**

Intracranial hemorrhage is defined as an acute or subacute episode of bleeding within the intracranial space, including hemorrhagic stroke, subdural hemorrhage and epidural hemorrhage.

**Major bleeding**

Major bleeding was defined as any episode of fatal bleeding event, bleeding that occurred in the critical sites (intracranial, intra-articular or intramuscular with compartment syndrome, intraocular, pericardial, retroperitoneal), bleeding necessitating transfusion, or bleeding that caused a drop in hemoglobin of ≥2g/dL, in accordance to the International Society on Thrombosis and Hemostasis.

**BASELINE VARIABLES DEFINITIONS**

**Estimated glomerular filtration rate**

Estimated glomerular filtration rate (eGFR) is calculated based on MDRD equation, expressed as:

186 x (Creatinine/88.4) - 1.154 x (Age) - 0.203 x (0.742 if female) x (1.210 if black)

where Creatinine is expressed in μmol/L.

**Anemia**

Anemia is defined as hemoglobin <13g/dL for men and hemoglobin <12g/dL for women.

Figure

Supplemental Figure 1. Calibration plot of CARDIAC score.


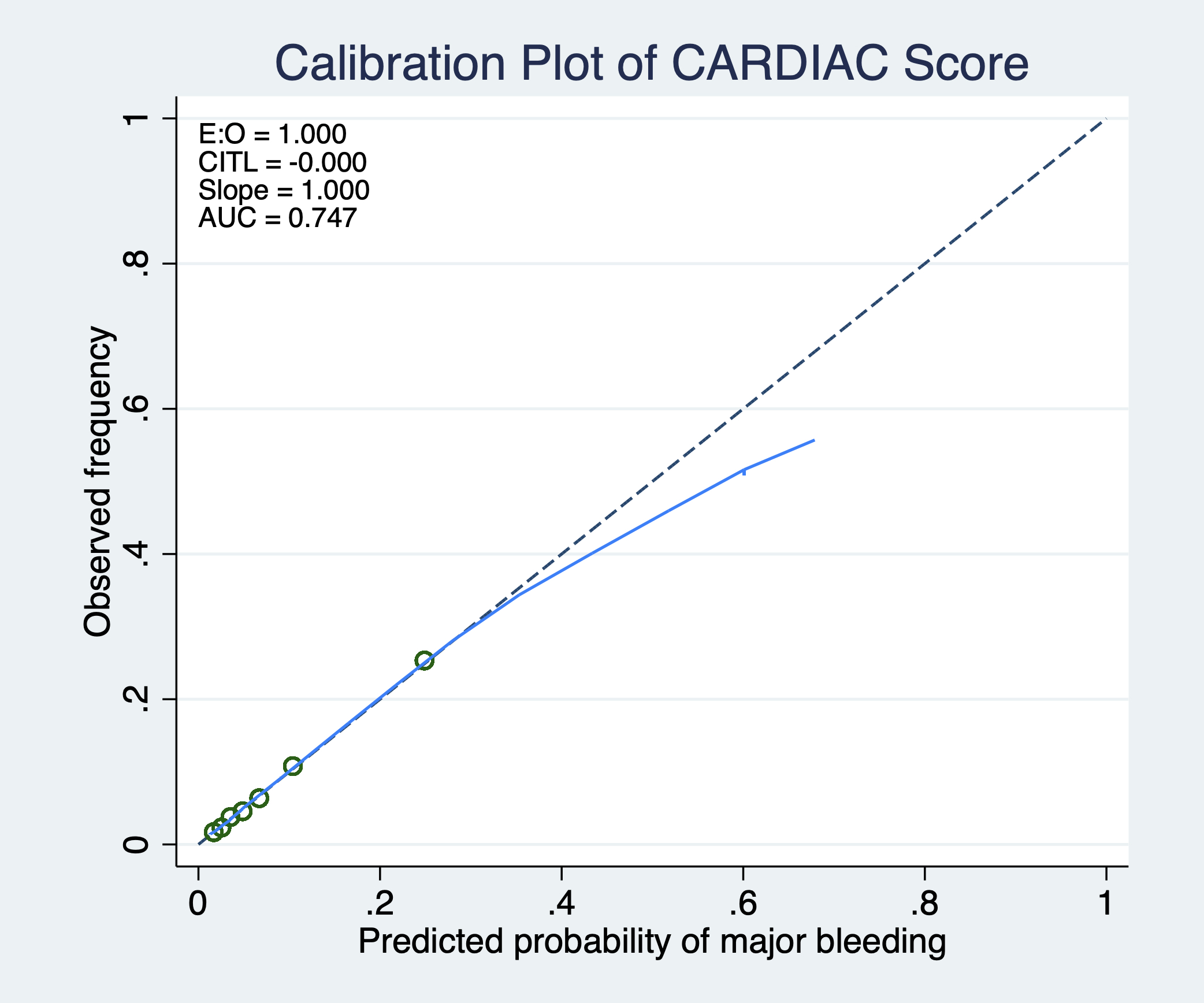


Tables

Supplemental Table 1. Types of bleeding between hospital discharge and 1 year after PCI. Numbers add up to over 100% due to overlapping.

| Types of bleeding | Number | Percentage (out of all bleeding events) |
| --- | --- | --- |
| Gastrointestinal bleeding | 626 | 34.6% |
| Intracranial hemorrhage | 189 | 10.5% |
| Intraocular bleeding | 95 | 5.3% |
| Intraarticular bleeding | 7 | 0.4% |
| Bleeding with hemoglobin drop >2g/dL or requiring transfusion of ≥2 units of blood product | 1276 | 70.6% |
| Fatal bleeding | 68 | 3.8% |
| Any major bleeding | 1808 | 100% |

| Outcomes | Hazard Ratio  (95% CI) | P value |
| --- | --- | --- |
|  |  |  |
| Primary |  |  |
| All-cause mortality | 2.10  (1.88 – 2.37) | <0.001 |
| Cardiovascular mortality | 1.84  (1.49 – 2.28) | <0.001 |
|  |  |  |
| Secondary |  |  |
| Major adverse cardiac events | 2.10  (1.94 – 2.28) | <0.001 |
| Myocardial infarction | 1.78  (1.58 – 2.00) | <0.001 |
| Unplanned revascularization | 2.12  (1.86 – 2.43) | <0.001 |
| Stroke | 2.30  (1.98 – 2.67) | <0.001 |
| Ischemic stroke | 1.52  (1.25 – 1.84) | <0.001 |
| Hemorrhagic stroke | 5.84  (4.70 – 7.26) | <0.001 |

Supplemental Table 2. Adjusted hazard ratios of outcomes using mixed effect model to adjust for the calendar year which PCI was performed.

Supplemental Table 3. Independent predictors of major bleeding between hospital discharge and 1 year after PCI in the development cohort using backward stepwise logistic regression and final risk score calculation.

| Variable | β | Odds ratio (95% CI) | P value | Points assignment^ |
| --- | --- | --- | --- | --- |
| Anti-Coagulation therapy | 0.676 | 1.97 (1.46 – 2.64) | <0.001 | 0 or 2 |
| Age group (every 10 years above 50 until >80) | 0.154 | 1.17 (1.09 – 1.25) | <0.001 | 0 – 4 |
| Renal insufficiency (each stage of CKD) | 0.171 | 1.19 (1.10 – 1.28) | <0.001 | 0 – 4 |
| Drop In hemoglobin (every g/dL drop) | 0.418 | 1.52 (1.43 – 1.61) | <0.001 | 0 – 10 in our cohort |
| Anemia at baseline (every g/dL below 12) | 0.601 | 1.82 (1.69 – 1.97) | <0.001 | 0 – 8 in our cohort |

^Final risk score, by the name of CARDIAC score, calculated by adding up points assigned for each variable as appropriate.

^In our cohort, risk score ranged from 0 to 16. A risk score of ≥5 had the best discriminating power for prediction of major bleeding.

Abbreviations: CI, confidence interval; PCI, percutaneous coronary intervention

Supplemental Table 4. Absolute risk of major bleeding with in the first year after percutaneous coronary intervention as predicted by the CARDIAC score.

| CARDIAC score | All patients | Development cohort | Validation cohort |
| --- | --- | --- | --- |
| 0 | 1.5% | 1.0% | 2.0% |
| 1 | 1.8% | 1.8% | 1.7% |
| 2 | 2.3% | 2.1% | 2.6% |
| 3 | 3.8% | 3.9% | 3.6% |
| 4 | 4.6% | 4.6% | 4.5% |
| 5 | 6.4% | 5.3% | 7.4% |
| 6 | 10.3% | 10.9% | 9.6% |
| 7 | 11.4% | 13.6% | 9.1% |
| 8 | 16.9% | 16.7% | 17.2% |
| 9 | 25.1% | 25.3% | 24.9% |
| 10 | 29.6% | 30.5% | 28.6% |
| 11 | 32.4% | 30.8% | 34.0% |
| 12 | 41.4% | 44.2% | 38.8% |
| 13 | 42.1% | 40.0% | 43.8% |
| 14 | 40.0% | 37.5% | 50.0% |
| 15 | 40.0% | N/A | 100.0% |
